# Regeneration of Dermis: Scarring and Cells Involved

**DOI:** 10.3390/cells8060607

**Published:** 2019-06-18

**Authors:** Alexandra L. Rippa, Ekaterina P. Kalabusheva, Ekaterina A. Vorotelyak

**Affiliations:** 1 Laboratory of Cell Biology, N.K. Koltsov Institute of Developmental Biology, 26 Vavilov str., Moscow 119334, Russia; rippa86@yandex.ru (A.L.R.); kalabusheva.e@gmail.com (E.P.K.); 2 Department of Regenerative Medicine, Pirogov Russian National Research Medical University, 1 Ostrovityanova, Moscow 117997, Russia; 3 Department of Cell Biology and Histology, Biological Faculty, Lomonosov Moscow State University, 1 Leninskiye gory, Moscow 119234, Russia

**Keywords:** skin, fibroblasts, myofibroblasts, wound healing, regeneration, scarring, keloid, skin substitutes

## Abstract

There are many studies on certain skin cell specifications and their contribution to wound healing. In this review, we provide an overview of dermal cell heterogeneity and their participation in skin repair, scar formation, and in the composition of skin substitutes. The papillary, reticular, and hair follicle associated fibroblasts differ not only topographically, but also functionally. Human skin has a number of particular characteristics that are different from murine skin. This should be taken into account in experimental procedures. Dermal cells react differently to skin wounding, remodel the extracellular matrix in their own manner, and convert to myofibroblasts to different extents. Recent studies indicate a special role of papillary fibroblasts in the favorable outcome of wound healing and epithelial-mesenchyme interactions. Neofolliculogenesis can substantially reduce scarring. The role of hair follicle mesenchyme cells in skin repair and possible therapeutic applications is discussed. Participation of dermal cell types in wound healing is described, with the addition of possible mechanisms underlying different outcomes in embryonic and adult tissues in the context of cell population characteristics and extracellular matrix composition and properties. Dermal white adipose tissue involvement in wound healing is also overviewed. Characteristics of myofibroblasts and their activity in scar formation is extensively discussed. Cellular mechanisms of scarring and possible ways for its prevention are highlighted. Data on keloid cells are provided with emphasis on their specific characteristics. We also discuss the contribution of tissue tension to the scar formation as well as the criteria and effectiveness of skin substitutes in skin reconstruction. Special attention is given to the properties of skin substitutes in terms of cell composition and the ability to prevent scarring.

## 1. Dermis Structure and Composition

The dermis is the mesenchymal component of the skin, separated from the epidermis by the basement membrane. The dermis comprises two structurally different layers named the papillary and reticular layer. The papillary layer, which is located closer to the skin surface, reaches a width of 300–400 microns, depending on the age and location. In the upper part, it is organized into cords, which are called dermal papillae that contain nerve endings [1] and microvascular vessels [2], necessary for nourishment and innervation. Papillary dermis differs from the reticular by a higher density of cells [3], a higher content of proteoglycans [4], and a weaker alignment of collagen fibers [5]. The papillary dermis has an uneven polar structure: its density decreases in the direction from the basement membrane to the reticular dermis [6].

**Figure 1:**
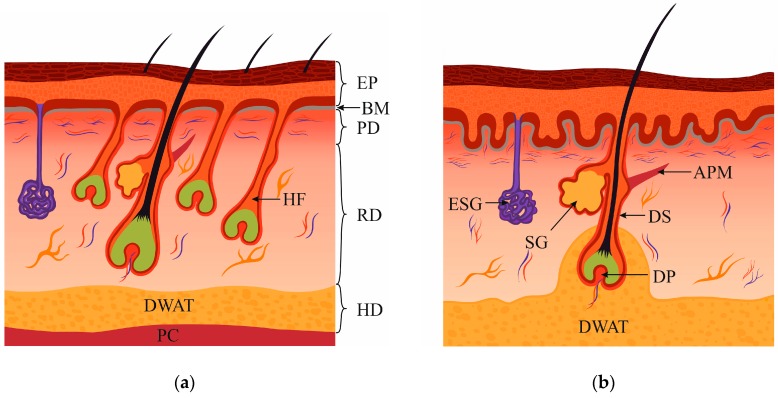
Skin structure: (**a**) murine skin structure. The murine skin has a high density of HFs including tylotrich and non-tylotrich HFs. The mouse back skin has four types including guard (tylotrich) and awl, auchene, zigzag HFs (non-tylotrich). The panniculus carnosus is under the hypoderm. (**b**) Human skin structure. Human skin structure differs from that of the murine. Epidermis is thicker and forms ingrowths called rete ridges. Accordingly, the papillary dermis forms dermal papillae. Two types of HFs are distinguished: guard HFs and vellus HFs. HFs density in the skin is less as compared to mouse one. DWAT is cone shaped. APM—arrector pili muscle; BM—basement membrane; DP—dermal papillae; DS—dermal sheath; DWAT—dermal white adipose tissue; EP—epidermis; ESG—eccrine sweat gland; HD—hypodermis; HF—hair follicle; PC—panniculus carnosus; PD—papillary dermis; RD—reticular dermis; SG—sebaceous gland.

The reticular dermis is separated from the papillary by the vascular plexus, rete subpapillare. The extracellular matrix (ECM) of the reticular dermis has a more pronounced structure: collagen bundles are organized into dense fibers, which, together with elastin strands, create an ordered network [7]. With aging, the papillary dermis decreases in volume, becomes thinner, and is gradually replaced by the reticular [3]. In general, human skin structure differs from that of the murine skin (Figure 1a,b). This fact should always be kept in mind in experimental studies.

Fibroblasts (FBs) are the most abundant cells in the dermis. A characteristic feature of these cells is the ability to synthesize and remodel ECM. Remodeling is supported by the synthesis of the cleaving metalloproteinases and their inhibitors. The ability to synthesize collagen I is the main and unifying typical feature of FBs [8]. A set of markers that characterize various sub-populations of FBs has been identified [9]. Pan-fibroblast markers CD90 [10], PDGFRα, and PDGFRβ [9] as well as the small leucine-rich proteoglycans, decorin and lumican, that regulate the assembly of collagen fibrils [11] are expressed at a high level in FBs throughout the dermis both in vivo and in vitro. Single-cell transcriptional profiling of human FBs showed that CD90+ FBs constitute only a small part of all cells within the dermis. The latter are represented mainly by CD31+ endothelial cells, CD45+ hematopoietic cells, neuronal cells, HF keratinocytes, and sweat gland cells [11]. FBs of the dermis are a heterogeneous population of cells, the specificity of which is determined mainly by their location relative to the layers of the dermis. In the papillary layer, there are more FBs with high enzymatic activity when compared to the reticular layer [12]. Significant differences between them are observed in the expression of specific components of ECM and markers, both in the organism and in culture [11,13,14]. In human skin, Tabib et al. identified major FB populations distinguished by the expression of SFRP2 and FMO1, which were additionally positive for DPP4 and LSP1, respectively, both at the RNA and protein levels [15]. SFRP2+ FBs are small, elongated, and distributed between collagen bundles, while FMO1+ FBs are larger and distributed both in the interstitial and in the perivascular space. In a recent study, Korosec and colleagues found two cell surface markers that distinguished the papillary and reticular FBs of the human dermis. Flow cytometric analysis of FBs isolated from the superficial and lower layers of the dermis showed that FAP+CD90– cells are enriched in the papillary dermis. Papillary FBs exhibit increased proliferative potential, express PDPN and NTN1 [13], and cannot be differentiated into adipocytes. FAP–CD90+ FB express high levels of ACTA2, MGP, PPARγ, and CD36 and easily undergo adipogenic differentiation, which is a hallmark of reticular FBs [16].

## 2. Papillary Fibroblasts

FBs of the papillary layer retain increased proliferative and synthetic activity in culture [12,13,17]. It has been shown that they synthesize more proteoglycans, but less collagen when compared with FBs of the reticular layer [18,19]. It was previously thought that papillary FBs are not particularly involved in the synthesis of ECM [20], however, it has recently been shown that matrix derived from papillary FBs rather than from reticular FBs increase keratinocyte viability. In addition, the longevity of the epidermis increased when cultured on papillary fibroblast-derived matrix skin equivalents when compared with reticular-derived matrix skin equivalents. ECM components specific to the papillary dermis may explain the predominant growth of keratinocytes on the papillary dermis [21]. In culture, FBs of the papillary layer have a spindle-shaped morphology [11]. The older the FB donor, the more spread out these cells are on the substrate [22]. With age, the papillary FB population loses its high growth potential and high proportion of small cells with a low degree of granularity. The papillary FBs from younger donors supported the formation of a more stratified and differentiated epidermis than the reticular FBs of a similar age. Interestingly, upon transplantation of these reconstructed skin samples to nude mice, only those that contained young papillary FBs formed rete ridge-like structures. Importantly, all of these features in young papillary FBs were markedly reduced in papillary FBs from old donors [3].

A small part of the cells of the FBs’ papillary layer is positive when stained for α-SMA. Papillary FBs have specifically increased expression of proteins responsible for the immune response [13]. Based on the expression of CD26 (Dpp4), Dlk1, and Sca1 [14], Philippeos and colleagues isolated the population of CD26+ Sca1– papillary FBs in the mouse dermis. These cells were distinguished by the expression of the COL6A5, COL23A1, WNT signaling genes (WIF1, APCDD1, RSP, and AXIN2), APCDD1, HSPB3, CD3g, CD3d, CD3ε, and CD39 [11]. However, expression of the WNT pathway proteins was lost in the culture conditions as it apparently requires the preservation of the niche including interaction with keratinocytes to function. However, it is the papillary FBs that support the formation of the basement membrane and the stratified epidermis in 3D culture with keratinocytes [13,23,24]. CD39 seems to be a conservative marker of papillary dermis in both human and mouse cells. The colonization of cell-free dermis by human papillary CD31–CD45–Ecad–CD90+CD39+ FBs with the addition of keratinocytes led to the formation of multilayered epidermis and epidermal ridges, unlike that of the reticular FBs. In the papillary dermis, the level of the WNT signaling is increased in both mouse and human cells [11].

Interactions between the epithelium and mesenchyme are critically important for the development and regeneration of the epidermis. Both sides release signaling factors that regulate cellular behavior in a reciprocal manner. Sorrell and colleagues showed the differences in the release of such critical signaling factors in the regulation of keratinocyte physiology as KGF and GM-CSF by FBs. In culture, papillary FBs synthesize less KGF when compared with reticular FBs. This difference persists even when FBs are exposed to IL1 and TNFα (proinflammatory cytokines, which are known to be released by keratinocytes) and keratinocyte conditioned medium. Cultured papillary FBs are characterized by an increased expression of GM-CSF upon stimulation, although expression of this factor is the same for cells of both layers of the dermis in vivo. In addition, papillary and reticular FBs produced approximately equal amounts of IL-6. This suggests that the production of IL-6 may not be a significant factor in the differential interactions between subpopulations of FBs and keratinocytes [23]. In the course of long-term cultivation, FBs of the papillary layer acquire the reticular phenotype and morphology [25]. A similar effect can be achieved by exposure to TGF-β, however, papillary FBs become refractory to its effects in the presence of keratinocytes [26].

## 3. Reticular Fibroblasts

The FBs of the reticular layer are usually strongly spread out over the substrate and have a stellate shape [22]. Actin fibers are diffuse [3]. In addition, most of the FBs of the reticular layer in culture are positive for α-SMA [13,19]. Studies have shown that this cell population has an increased expression of genes responsible for cell mobility and contraction. MGP was reported to be a specific marker for the FBs of the reticular layer [13]. TGM2 expression has also been noted in the reticular FBs population [13]. TGM2 is associated with the activation of TGF-β1 [27]. Moreover, the reticular FBs themselves produce TGF-β1 more intensively [28]. TGF-β1 is associated with matrix production and differentiation of myofibroblasts (mFBs). This is one of the explanations for properties of the reticular FBs population, such as high matrix density and an increased number of α-SMA-positive cells [13]. Philippeos and colleagues identified subpopulations of the lower mouse dermis FBs including Dlk1+Sca1– reticular FBs and two subpopulations of preadipocytes including Dlk1+Sca1+ and Dlk1–Sca1+. Mouse reticular FBs expressed genes of the secretoglobin superfamily (SCGB2A2, SCGB1D2, and SLC12A2). CD36 was upregulated in the lower reticular dermis and hypodermis. Gene ontology analysis of differentially expressing genes showed genes related to ECM organization were predominant for reticular FBs, muscle linkage for Dlk–Sca1+ preadipocytes, chemotaxis, and inflammation for the Dlk+Sca1+ preadipocytes. At the same time, it has been shown that Dlk+Sca1+ preadipocytes, unlike Dlk–Sca1+ preadipocytes, express genes encoding fibrillar proteins ECM at a high level. However, both populations expressed preadipocyte marker proteins at a high level. Single-cell RNA sequencing of human FBs determined a distinctive expression of CD26, MFAP5, PRG4, and the lack of expression of COL6A5 in reticular FBs. The authors found that CD26 was absent in the uppermost papillary part of the adult human dermis, although it was widespread in the rest of the dermis [11]. At the same time, CD26 marks the superior papillary dermis at the early stages of mouse development [14] and in an adult mouse, expression of CD26 is found in a large fraction of dermal FBs [29].

The effect of the FBs of the reticular layer on keratinocytes is due to the increased synthesis of KGF and reduced GM-CSF relative to the FBs of the papillary layer. Keratinocytes cultured with reticular FBs have an irregular shape, and synthesize specific markers of the basal and intermediate layers, but not the terminal one. The composition of the basement membrane is altered [23]. When the acellular dermis is populated with human reticular FBs, a fully stratified epidermis is not formed [11].

Characteristics of papillary versus reticular FBs are presented in Table 1.

## 4. Fibroblasts Associated with Hair Follicles

The HF consists of epithelial and mesenchymal parts. The mesenchymal compartment is represented by the dermal papilla and its derivative connective tissue sheath. The dermal papilla is located at the base of the HF and makes contact with the connective tissue sheath through a thin isthmus. Studies have shown that both dermal papilla and the connective tissue sheath contain mesenchymal stem cells [31,32,33]. This fact has placed the dermis in line with other organs containing mesenchymal stem cells such as adipose tissue or bone marrow, and has forced the attention of researchers as one of the potential sources of material for cell therapy. Currently, this area is actively developing in relation to the treatment of skin diseases of various origins [34].

Dermal FBs are the most commonly used cells in the composition of living SS. The use of dermal papilla cells shows that they reorganize the matrix to a similar extent as FBs in orienting collagen fibers [35], but their application reduces the risk of fibrotic processes associated with wound healing [36]. Cells of the dermal papilla and connective tissue sheath support keratinocyte differentiation and stratification in skin equivalents similarly to dermal FBs [37], while the cells of the connective tissue sheath stimulate the formation of a denser basement membrane. The use of the medium conditioned by the cells of the connective tissue sheath in the treatment of diabetic wounds also contributes to the formation of a more developed layer of the epidermis in comparison with the effect exerted by mesenchymal stem cells of the bone marrow, although it does not affect the rate of wound healing [38]. Unpublished data obtained in our laboratory show that dermal papilla cells can be used in cell therapy of diabetic wounds instead of mesenchymal cells of bone marrow and adipose tissue, since HF avoids the negative effect of the diabetic environment in contrast to adipose tissue and bone marrow.

However, the main interest in the use of dermal papilla cells and the lower part of the connective tissue sheath has focused on their unique ability to stimulate HF regeneration [39,40]. Cells migrate between these mesenchymal structures during the HF cycle, and it is the migratory population that possesses the characteristics of stem cells capable of stimulating the neogenesis of HF and its mesenchymal compartments [41]. Researchers have focused both on the reconstruction of individual HFs [42,43,44,45] and on obtaining living SS with HFs [46,47,48]. It should be noted that the formation of HFs in wounds has antifibrotic and angiogenic effect [49,50].

In the skin, vascularization of HFs changes during the cycle: an increase in the lumen of the blood vessels is necessary for HF during the active growth phase [51]. Changes in angiogenesis regulate both HF epithelial cells [52] and dermal papilla cells [53]. The latter are able to interact with the endothelium of the microvasculature, supporting its proliferation, functioning, and stimulating of tubulogenesis in culture conditions [54].

The antifibrotic effect of the HFs is due to the BMP family of proteins, which are responsible for the development and regulation of the HF cycle. HFs retain their anti-fibrotic potential in culture [49], which indicates that the dermal papilla inside HF produces this effect due to its activity in BMP expression.

The combination of epidermal keratinocytes and adult human dermal papilla cells in the living SS does not allow for the development of fully functional and properly structured HFs. However, the use of specialized trichogenic epidermal stem cells instead of keratinocytes promoted the development of rudimentary hair follicles in vivo. This effect was sufficient to prevent the formation of scars and stimulate vascular growth [46].

Summarizing the positive effects of HFs in wounds, it can be suggested that neofolliculogenesis is a natural course of normal regeneration versus fibrosis or chronic wounds. In humans, HFs in the wound area can appear only by transplantation of mature follicles, or by using tissue-engineered constructs bearing the rudiments or mature HFs. Nevertheless, the results of experiments with genetic modifications on laboratory animals allow us to expect progress in this area. The purpose of these experiments was to stimulate neofolliculogenesis in adult mice. The morphogenesis of HF and dermal papilla is specifically regulated by members of the WNT, FGF, TGF-β, SHH, and other families. The main event during the formation of the dermal papilla is the activation of WNT signaling in the epithelial placode, which causes FGF20, which, in turn, is responsible for the condensation of FBs and dermal condensate formation [55,56]. Another important factor in the development of the dermal papilla is SHH; knockout of its signaling in the dermis prevents the formation of fully developed HFs [57]. Activating WNT/β-catenin signaling in the epidermis and SHH in the dermis triggers the formation of new HFs in adult mice [50,58].

It is worth noting the significant prospects in the application of mesenchymal HF cells in cell therapy. To date, there are no reliable signs of direct involvement of these cells in the processes of post-traumatic wound healing in vivo [59], however, these cells prevent fibrotic effects, and promote angiogenesis and development of the epidermis. The researchers noted that if you needed to create a cellular construct aimed at regenerating the skin “in general” and quickly closing the wound area, it was better to use cells of the connective tissue sheath, since dermal papilla cells are likely to be more differentiated in the direction of controlling the HF cycle [37]. At the same time, precisely due to this high specialization of dermal papilla cells, their use in the reconstruction of the entire skin structure and the normalization of its physiological features is possible due to the formation of mature functioning HFs.

## 5. Dermal Cells Participating in Different Modes of Wound Healing

Wound healing in skin depends on the coordinated collective activity of several cell types. Keratinocytes and FBs migrate into the wound to restore skin structure. Furthermore, other cells participate in the process including hemopoietic, endothelial, immune, neural, white fat cells, pericytes, Schwann cells, tissue-specific stem cells, and their progenitors. FBs play a pivotal role in wound healing, mediate fibrosis, participate in inflammatory networks, synthesize matrix, and modulate immune cell functions.

Shortly after wounding, the wound is populated with FBs from the reticular dermis and cells from the hypodermis [14]. FBs of the reticular dermis are able to produce thick and well-organized collagen fibers in contrast to papillary FBs that are attracted later (during epithelialization) and synthesize poorly organized ECM. Reticular FBs generate a collagen matrix typical for fibrosis [14,60,61]. Immediate participation of reticular FBs in regeneration may explain the absence of HFs in the newly formed scar tissue, as it has been demonstrated that these FB compartments do not contribute to HF mesenchymal components during development, unlike papillary FBs. WNT/β-catenin signaling plays a key and complex role in both the ability of HF formation at the wound site and in the development of fibrosis. It should be noted that the loss of the ability of hair formation in adult skin probably does not reflect the lack of ability of the epidermis to form new HFs. In mice, ectopic HFs can be induced in the skin with or without injury using epidermal activation of β-catenin [62,63,64,65]. It has been shown that β-catenin activation in the epidermis activates both reticular and papillary FBs. However, only stimulation of the papillary dermis permits new HF formation [14]. Herewith, epidermal induction of β-catenin in functionally different papillary and reticular FBs is transduced through different signaling pathways [58]. It was previously shown that β-catenin signaling mediates fibrosis [66,67,68,69,70]. It has recently been shown that inhibition of β-catenin signaling in dermal FBs enhances the regeneration of HFs during wound healing. Postnatal β-catenin ablation in FBs contributed to HF regeneration in neonatal (1.52-fold) and adult (3.27-fold) mouse wounds, while β-catenin activation reduced HF regeneration in neonatal wounds by 30%, and had no effect on HF regeneration in adult mouse wounds [71]. The results of Rognoni and colleagues reflect a model where the lack of ability to form HFs by postnatal wounded skin is associated with an increase in the activation of WNT/β-catenin signaling in the wound bed, which in turn increases the number of reticular FBs unable to induce HF formation. In studies directed toward determining differences in reticular and papillary FB responses to injury and induction of HF regeneration, the role of WNT signaling and its targets in this process has a great impact on overcoming the problems of fibrosis and the absence of skin appendages in the regenerated tissue. When injured, the density of papillary FBs decreases and the β-catenin activity in the dermis increases with age, which is linked to an increase in reticular FB expansion. In this case, the expansion of FBs takes place with minimal proliferation. Repair of postnatal skin wounds occurs quickly by attracting reticular FBs, but at the cost of losing the regeneration of HFs at the wound site [71]. It can be supposed that normal epithelial–mesenchymal interactions occur between the epidermis and the upper layer of the dermis represented by its papillary part, and maintenance of this interaction is of vital importance for scarless healing and the generation of new appendages. Reticular FBs fail to provide proper clues for skin morphogenesis. Moreover, in the wound, they organize the matrix typical of that for fibrosis. Therefore, it is the papillary dermis that may provide reparative regeneration of the skin. Interestingly, in clinical practice, surgeons have learnt to destroy the upper layer of the skin down to the interface between the papillary and reticular dermis to improve conditions such as acne scars. However, it is known that one should not go deeper in order to prevent permanent scarring [30].

Rapid repopulation of the wound with FBs and ECM synthesis causes scar formation in adults in contrast to embryos [72,73]. Scarless healing of fetal tissue depends on wound size and gestational age [74]. It is known that immune responses during prenatal development are weakened when compared to that in the adult body. In addition, expression profiles of ECM proteins, growth factors, and cytokines in the damaged area differ significantly [72,75]. For example, differences in the expression of PDGF, FGFs, VEGF, and TGF-β were found [76]. TGF-β is thought to play the key role. In wounds, three isoforms of this growth factor are detected: TGF-β1, TGF-β2, and TGF-β3. The first two isoforms are thought to be profibrotic, while the latter is antifibrotic. When adult skin is injured, the first two isoforms predominate, while the antifibrotic isoform prevails in damaged embryonic skin [77]. Fetal and postnatal FBs respond differently to TGF-β. In the presence of TGF-β, postnatal FBs increase their proliferation, in contrast to embryonic cells [78,79]. The difference in cell response under the influence of TGF-β1 was shown, and stimulates the production of COL1A1, CTGF, PAI1, TGF-β1, and TGF-β3 in postnatal FBs. Fetal FBs respond to TGF-β1 by increasing the expression of COL3A1, PAI1, TGF-β2, a delayed upregulation of TGF-β3, and decreasing the expression of COL1A1, CTGF, and TGF-β1 [80]. It was noted that therapeutic agents based on the neutralization of PDGF, TGF-β1, and TGF-β2 or the addition of exogenous TGF-β3 markedly reduced or prevented scarring [81]. Phase I and II clinical studies of recombinant human TGF-β3 (Avotermin or Juvista) have been successful, where the administration of TGF-β3 reduced scars [82,83]. However, phase III failed and, among other things, the correct system of standardization of the amount of active substance in the sample during the production process was missed [84].

Collagen I is the dominant component of the ECM in the dermis and accounts for approximately 70% of its dry weight. Moreover, in intact adult skin, the ratio of collagen I to collagen III is approximately 4:1, whereas it is approximately 1:1 in neonatal skin. In mature skin, less hyaluronic acid (HA) is secreted. The amount of collagen III temporarily increases when the skin is injured and during the formation of neodermis. In newly healed human skin, the ratio of collagen I and III is about 1:1, as it is in neonatal skin. At the same time, in response to a wound, fetal skin shows a higher amount of collagen III and HA, and lower collagen I [30,85].

HA is the main component of the fetal ECM, it stimulates the migration of FBs. Unlike adult FBs, fetal FBs do not reduce HA synthesis with an increase in cell density [86]. The HA-based matrix is effective as a regenerative skin matrix [87,88,89,90]. In addition to the high content of collagen III and HA in the fetal dermis, there is plenty of fibronectin and a rapid deposition of tenascin in fetal wounds, which provides rapid deposition of the matrix and early wound epithelization [75]. It is also assumed that the composition of the ECM of embryonic skin has reduced stiffness and thereby contributes to scarless regeneration [91].

Fetal FBs migrate faster than adult cells. Their increased migration rate during wound healing has an impact on collagen deposition. Fetal FBs exhibit a unique contractile phenotype that facilitates ECM assembly in a manner that re-establishes the natural structure and function in the dermis. This cellular behavior is preserved in vitro and in transplantation experiments in vivo [92]. It was recently demonstrated that this phenotype of fetal FBs impedes their differentiation into mFBs under the influence of TGF-β1 [93].

Understanding the regulation mechanisms of the phenotypic characteristics of fetal FBs that allow them to not transform into mature mFBs in wound conditions and properly synthesize ECM, may lead to new therapeutic targets oriented toward reducing scar formation and fibrosis. In addition, it has recently been shown that fetal skin proteins are able to induce HF neogenesis. A proteomic analysis revealed three secreted embryonic skin proteins that were sufficient to induce new HFs, namely apolipoprotein-A1, galectin-1, and lumican. The treatment of mature FBs incapable of forming HFs with cell-free extracts from embryonic skin or a combination of the three proteins above-mentioned altered gene expression in FBs to the gene profile of hair-inducing dermal papilla cells with the activation of IGF and WNT signaling. The authors noted that the combination of these proteins in dermal skin equivalents could help create trichogenic skin substitutes (SS) [94].

**Scheme 1:**
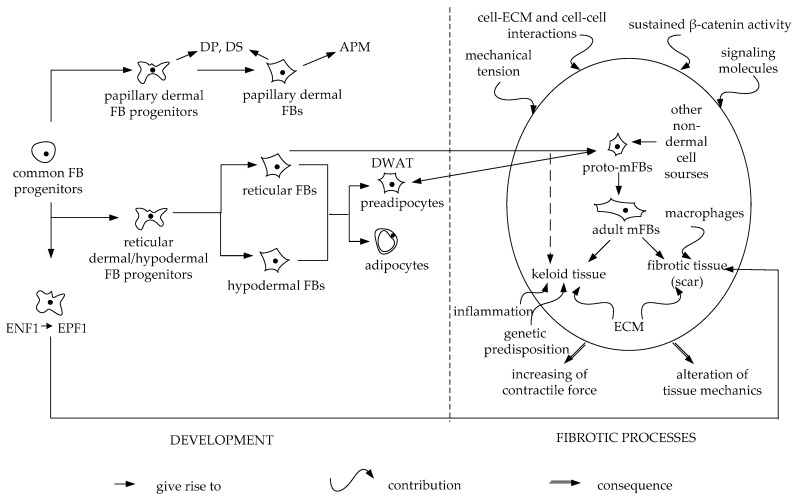
Origin of dermal cell types and their participation in fibrosis. The left part of the scheme is a schematic representation of dermal cells development and their heterogeneity, using data from Driskell with co-authors [14] with the addition of data from Jiang with co-authors [95] on the participation of ENF1 and EPF1 cells in the scar formation. The right part of the scheme summarizes data on the relationship of cellular and other factors on scar formation. APM—arrector pili muscle; DP—dermal papilla; DS—dermal sheath; DWAT—dermal white adipose tissue; ECM—extracellular matrix; ENFs—engrailed 1-history-naive fibroblasts; EPFs—engrailed 1-history-positive fibroblasts; FB—fibroblast; mFB—myofibroblatst.

Studies performed to determine the origin of dermal cells in the early development of a mouse embryo showed that a common mesenchymal precursor is detected before E12.5. Further on, FBs determination occurs: progenitor papillary FBs and reticular FBs form from a common precursor. Further in development, progenitor FBs of the papillary dermis give rise to dermal papilla cells and arrector pili muscles, while progenitor FBs of the reticular dermis give rise to progenitor adipocytes (Scheme 1) [9,14].

Recent study has shown the presence of a subpopulation of embryonic FBs that are responsible for future scar formation. These somitic-derived FBs express Engrailed 1 [95]. Engrailed are homeobox genes that participate in the regulation of development in the dorsal midbrain and anterior hindbrain. In mouse, after E9.5, Engrailed 1 is expressed in somites and limb ectoderm throughout development [96]. It was shown that Engrailed 1-history-naive fibroblasts (ENFs) are responsible for dermis regeneration, the number of which is critically reduced with fetus development. ENFs are replaced by Engrailed 1-history-positive fibroblasts (EPFs), the expansion of which subsequently leads to scar formation (Scheme 1). At the same time, both types of these cells (EPFs and ENFs) are not predominantly localized in either the papillary or the reticular dermis. ENFs deposit fibronectin, generating a preliminary matrix and then are replaced with EPFs, which produce mature dermis and deposit collagen I and collagen III. Thus, the development of the dermal lattice suggests cellular conversion of ENFs to EPFs [95].

In the early stages of embryogenesis, when a common mesenchymal precursor is detected [14], ENFs regenerate a wound without EPFs interference and a scar is not formed. The transition from scar-free healing to scar formation occurs after an increase in EPFs abundance in the course of dermis embryogenesis. The ENF-to-EPF switch in dermal embryogenesis explains the transition from regeneration to scarring [95]. Interestingly, after somitic-derived EPFs transplantation into the oral cavity, these cells, regardless of the environment, deposit dense collagen fibrils, demonstrating ectopic connective tissue formation and the classic dorsal scar phenotype. After the transplantation of Wnt1 neural crest-derived FBs (which are responsible for connective tissue organization in the oral dermis) on the skin of the back, these cells showed significantly less scar development when compared to EPFs. This experiment demonstrates that wound healing does not depend on the local microenvironment of the tissue, but results mainly from innate cell properties. These data highlight the fact that the various lines of FBs in the skin have different roles in skin development and the response to injury. In addition, it seems that the main contribution to wound healing of the skin is made after all by certain resident FBs, but not by other mesenchymal or non-mesenchymal cell lines [29].

EPFs in mature skin express the surface marker CD26/DPP4. Inhibition of the enzymatic activity of CD26/DPP4 during wound healing leads to a reduction of scar formation in the mouse excisional skin wound. The use of CD26/DPP4 inhibitors has potential for clinical practice. Topical administration of CD26/DPP4 inhibitors in skin equivalents or dermal hydrogels may have a significant effect on scarless wound healing [29].

## 6. Keloid Scars and Keloid Cells

Scars differ from normal skin aesthetically, functionally, and morphologically. The scar is comprised of poorly structured and densely packed collagen fibers while the hair and glands are missing.

Keloids are abnormal scars caused by disturbances of skin wound healing characterized by excessive ECM production and are prone to form in areas of the body with increased skin tension or stiffness [97,98,99,100]. It is known that keloids do not have a malignant potential, but they cause physical ailments and affect the life quality of patients. The main clinical features of keloids are their progression beyond the original site of skin injury, persistence in time, and the absence of native regression [101,102]. Expanding, keloids cause itching, inflammation, pain, and psychological discomfort for patients, especially when keloid affects the face area. Currently, there are several methods for treating keloids, which act through different mechanisms and do not always provide a stable result. A better understanding of keloid pathogenesis will assist in the development of new and more effective treatments in the future [103]. Currently, there are no adequate models that can fully reproduce the nature of keloid tissue. Although keloids are human-specific, the appearance of a scar as a result of deep skin damage has been studied in animal models such as pigs, rabbits, mice, and guinea pigs [104]. In other studies, human keloid tissue has been transplanted to rodents. In the case of such xenotransplantations, the entire keloid environment in vivo is not fully reproduced [105,106,107]. An analysis of the monolayer culture of keloid FBs revealed their differences from normal FBs [108,109,110]. Keloid FBs are refractory to apoptosis, synthesize more ECM proteins, and express more cytokines [111]. There are in vitro models that study the behavior of keloid FBs inside a collagen gel [112,113,114]. Several studies compared the FBs profile from three locations: the center of the keloid, the periphery of the keloid, and normal skin surrounding the keloid in a monolayer cell culture [115,116]. In the work of Suttho and colleagues, the authors went further and tried to reproduce the 3D characteristics of keloid tissue in vitro, showing that the normal organization of the dermis (papillary and reticular one) with epidermal rete ridges was gradually lost during the transition from the periphery to the center of the keloid. In the center of the keloid, the preserved dermis structure resembled the reticular dermis. The authors suggest that FBs of the keloid center can only be reticular. The doubling time of FBs from the center of the keloid was significantly higher than that of FBs from the periphery of the scar or from intact skin [117]. These data correlate with other studies showing that the majority of FBs obtained from the periphery of the keloid were in a proliferative state, whereas FBs from the center of the keloid were usually in a resting phase [116]. FBs from the keloid center remodeled the matrix and contracted the gel quickly (within 24 h). Furthermore, their contractile ability ceased. The gel contraction by FBs from the center of the keloid was higher than that by the FBs from the periphery and normal tissue [117]. These results could be related to the especial histological structure of the keloid dermis. Recent study showed that in contrast to clearly distinguishable layers of the papillary and reticular dermis in normal skin, three layers of tissue were identified in the keloid dermis, differing in the structure of the ECM and the quantity of FBs. In addition, FBs from different layers of keloid dermis had different biological properties [118]. Presently, reliable models of human keloids and hypertrophic scars are urgently needed to promote studies of the keloid pathogenesis.

It was shown that in keloid tissue, in the epithelial cells of the epidermis and skin appendages, the expression of epithelial markers is lost and the expression of mesenchymal markers is enhanced [119]. This promotes aggressive growth and active invasion into normal tissue. Given the involvement of epithelial–mesenchymal transition (EMT) to the fibrotic wounds and the formation of hypertrophic scars [119,120,121,122], it is likely that the impact on the components of EMT signaling may alleviate fibrosis [123]. It is possible that the early and prolonged activation of EMT in pathological conditions in response to injury contributes to inflammation and fibrogenesis. In turn, prolonged unresolved inflammation and hypoxia conditions activate EMT in hypertrophic scars [120,121].

Keloid FBs have a high migratory activity and are able to migrate far beyond the initial wound. It has been shown that keloid FBs synthesize high levels of ECM. Keloid tissue is stiffer than the normal dermis, but at the same time, keloid FBs themselves are softer than normal FBs and show a loss of stiffness sensitivity. In keloid FBs, low expression of caveolin-1 (CAV1) has been detected [124]. CAV1, the main protein of caveolae envelope, is associated with the regulation of cellular mechanics. Low expression of CAV1 decreases cell rigidity, but increases contractile forces and the ability to migrate. In keloid FBs, decreased CAV1 increases the expression of the transcription factor RUNX2 responsible for osteogenesis, which is a potential regulator of increased ECM production in keloids and is associated with fibrogenesis [125]. In keloid FBs, COL11A1 is highly upregulated [125,126,127]. Interestingly, in normal skin, COL11A1 expression is low or absent, but in some types of cancer, COL11A1 is highly expressed [128,129]. Hsu et al. noted that keloid FBs have some mechanical properties like cancer cells including cell softening and the loss of stiffness sensing [125]. This work illustrated the importance of mechanical force in the keloid pathogenesis and an adequate response to it. Low CAV1 increases RUNX2 and alters cellular mechanics including cell softening, loss of stiffness sensitivity, and an increase in contractile force. Treatment of keloid with trichostatin A led to an increase in CAV1, elevated cell stiffness, and a decrease in their migratory ability [125]. When developing methods for treating keloid scars, one should probably focus on the scar not as a tissue mainly enriched with ECM, but by keloid cells, which determine the formation of a rough matrix. Perhaps there will be an effective therapy that changes the properties of keloid FBs and/or if the matrix is destroyed and the scar colonized with cells of a different type, it may be possible to overcome the pathological development of the scar, as new cells, both by their internal properties and by synthesizing ECM, can change the mechanical properties of keloid tissue. Relaxation of skin tissue tension has been successful in the treatment of keloids [99]. In the treatment of pathological scars, it is necessary to take into account that keloids and hypertrophic scars are the result of chronic inflammation in the reticular dermis and a number of treatments that reduce inflammation are actively used in the treatment of these pathologies [130].

## 7. Dermal White Adipose Tissue Involvement in Wound Healing

Recent studies have identified a new type of white adipose tissue, DWAT, which plays an important role in a variety of processes: wound healing, the immune response, HF homeostasis, and thermoregulation [131]. It has been shown that intradermal adipocytes are necessary and sufficient for HF stem cell activity. PDGF expression in immature adipocytes is involved in the regulation of follicular stem cell activity and activation of HF growth. Data from Festa et al. indicated that adipocyte lineage cells form the niche of epithelial stem cells, positively regulate their activity, and induce HF regeneration [132]. In mouse skin, DWAT is located between the dermis and panniculus carnosus (Figure 1a). In human skin, DWAT is deposited around single pilosebaceous units that include the HF bulb with a hair shaft, a sebaceous gland, and erector pili muscles, where DWAT engulfs HFs in the form of a cone (Figure 1b) [131]. Interestingly, in humans, the cone-shaped structures of DWAT are found only in areas of the body that are prone to scarring (for example, the abdomen, neck, chest, etc.) and is not pronounced in areas associated with reduced scarring (scalp, forehead, or early fetus) [133]. It is known that in mouse skin, DWAT changes its thickness during spontaneous and depilation-induced HF cycling [134]. DWAT cells have an impact on wound healing [135]. The absence of mature adipocytes in the skin [136] or inhibition of adipogenesis leads to impairment of the recruitment of FBs to the wound site and delayed wound closure [135]. Furthermore, the reprogramming of adipocytes from mFBs was detected during pathophysiological processes. Newly regenerated HFs in the mouse wound stimulated the reprogramming of the surrounding mFBs into preadipocytes, while such adipogenesis did not occur in hairless scar tissue. However, BMP signaling is necessary for the reprogramming of mFBs into preadipocytes since K14-Noggin mice, which overexpress Noggin, a soluble BMP antagonist, in the epithelial cells of HFs, and mice, which have the deleted BMP receptor BMPR1A, could not regenerate fat after injury despite the formation of the normal amount of HFs [49]. The study results indicate the field of opportunities for when and where it is possible to influence regeneration and prevent tissue scarring by activating the embryonic pathways and turning mFBs into adipocytes. The study showed that HFs grew independently of fat, but the HF regeneration and signaling pathways involved were necessary for the regeneration of skin fat. In this study, keloid scar cells were also treated with BMP4, which caused their conversion into adipocytes. Co-cultivation of keloid FBs with scalp HFs gave the same result. The authors believe that strategies for HF regeneration can ultimately benefit patients with scars and keloids, who have adipose tissue disorders and fat deficiency [49]. It can be assumed that the transplantation of adipocyte progenitors, HFs, or their components into the scar or keloid could significantly improve the scar tissue condition, both functionally and aesthetically. In a relevant 3D model of keloid in vitro, it will be possible to investigate the effect of such transplantations and signaling cascades responsible for regeneration.

It is reported that adiponectin-positive dermal progenitor cells are able to differentiate into mFBs. During the induction of fibrosis by bleomycin in mouse skin, adiponectin-positive precursors, whose presence is usually limited to DWAT, distributed over the entire damaged dermis over time, lost their adipocyte specific markers and expressed mFB markers [137]. Reduction of adipogenesis and the loss of DWAT are consistent features of cutaneous fibrosis. At the same time, DWAT can be replenished, since the regeneration of adipocytes from MFBs is possible [49]. On the other hand, the suppression or reversal of adipocyte transdifferentiation into mFBs, increase in adipocyte stem cells survival and their progeny, and antifibrotic cytokine expression may be effective directions of anti-fibrous therapy [137].

## 8. Myofibroblasts and Fibrosis

In skin repair, cells that are actively involved in the process and affect its outcome are mFBs. mFBs are first found in granulation tissue during skin wound healing as cells that have secretory characteristics of FBs and contractile functions that are similar to those of smooth muscle cells [138]. mFBs are defined as collagen secreting cells that have contractile ability and form microfilament bundles similar to stress fibers [139]. mFBs produce a variety of ECM proteins including fibronectin containing extra domain A (ED-A fibronectin), a large number of interstitial collagens, and hyaluronan [140,141,142]. Presence of a splice variant form of fibronectin, ED-A fibronectin, in surrounding ECM is a distinctive feature of mFBs which induces activity of these cells [143,144]. One characteristic of mFBs is its activation during wound healing and its absence in the majority of normal tissues [145]. It is widely accepted that when wound healing is completed in skin, mFBs undergo apoptosis [146], while they may be deactivated in other tissues in fibrosis [147,148,149]. It was recently shown that skin mFB spheroids are deactivated in experimental conditions [150]. It is difficult for mature mFBs to migrate and proliferate, since the cytoplasm is filled with a large number of contractile fibers [151]. Therefore, early activated and migrating FBs that lack a contractile apparatus and originate from resting tissue precursors are called proto-mFBs [152]. A distinctive feature of the mature mFB is the expression of α-SMA. However, not all α-SMA-expressing cells are mFBs such as smooth muscle cells, pericytes, myoepithelial cells, and endothelial cells [153,154]. These cells do not organize α-SMA into bundles of microfilaments, similar to stress fibers, which provides the most important specific function of ECM contraction for mFBs. At present, specific markers of mFBs have not been identified. Since mFBs can arise from different sources, a combination of positive and negative markers is required to identify mFBs in different tissues [155]. During skin repair, FBs were the first described population of cells that became mFBs [138]. Currently, we understand that skin FBs themselves are a heterogeneous population [8,11,14,29]. As in the case of mFBs, there is no exact and universal FB-specific marker that would clearly identify the type of FB from a variety of mesenchymal cells.

During the repair, mFBs are activated from various sources [156]. A series of mFB precursors was identified. The most well-known of them are resident FBs, epithelial and endothelial cells (via epithelial or endothelio-mesenchymal transition) [120], circulating fibrocytes derived from bone marrow [153,157], and pericytes [158,159,160]. Research findings suggest that mFB activation is not limited to just one progenitor cell. Therefore, the contribution of all potential precursors to the mFBs population complicates their definition and the presentation of the complete overview of repair. The process is complicated by mutual cell effects. For example, it has been shown that activation of the dermal FBs’ profibrotic profile can be affected by mesenchymal stem cells [161]. Thus, mFB is a state of cell activation that can be acquired by several cell types under specific conditions of tissue damage [156]. Sustained activation of mFBs leads to fibrous pathology in the skin and internal organs. mFBs are also known to be active in the stromal reaction of multiple types of cancer [153,162]. Scarring is part of the normal tissue repair response due to acute damage. Insufficient activation of mFBs can be observed in chronic wounds. Excessive and prolonged activation of mFBs lead to fibrosis. Sustained communication of inflammatory macrophages and mFBs remodeling the ECM is observed in fibrosis. The temporal and spatial coordination of the activities of these two cell populations is crucial for a controlled healing process. At the same time, ECM itself, as a repair coordinator, influences cellular processes and plays an important role in organizing the exchange of profibrotic signals between macrophages and mFBs [163]. mFBs are targets in the treatment of fibrosis, as well as the factors affecting it. The search for antifibrotic factors has shown that TGF-β3 suppresses scar formation [164,165]; bFGF (FGF-2) [166,167,168,169] and IL-1β [170,171] are suppressors of α-SMA expression and mFB generation, antagonizing the effect of TGF-β1. IFN-γ also suppresses α-SMA expression in FBs, collagen deposition, and contraction [172,173,174,175]. At the same time, inhibition of IFN-γ results in accelerated healing after burn injury [176]. The reduction of fibrosis is promoted by the inhibition of cytokines and factors affecting the activation of mFBs. In addition to profibrotic factors such as TGF-β1, CTGF, PDGF [155], IL-6 [177], and others, activation of mFBs from various precursors and increased fibrosis is affected by tissue mechanical tension [178]. Mature differentiated mFBs, due to an increase in mechanical tension, appear earlier in granulation tissue in full-thickness splint wounds when compared to normally healing wounds [179]. It has been shown that FBs cultured on substrates with different rigidity have different phenotypes [180]. Cultured on soft or compliant surfaces, FBs do not express stress fibrils. However, when the rigidity of the substrate increases, there is a dramatic change in the cells’ morphology and abundance of stress fibers [181]. Other mechanical signals, for example, created by the interstitial fluid flow are able to induce TGF-β1 production and thus differentiate cultured in collagen gel FBs, in the absence of other external mediators such as inflammatory environment [182]. In addition, pre-straining of the ECM regulates the bioavailability of TGF-β1 and transitions from the latent to the active form [183]. The hard matrix used in 3D cultures in vitro or in granulation tissue and fibrous tissues in vivo in combination with TGF-β1 stimulation can induce complete differentiation of mFBs [184]. It has been shown that the mechanical tension achieved by stretching the healing wound is sufficient for the formation of hypertrophic scars in mice. The mechanical load increased the activity of the mFBs’ cellular density and volume due to decreased cellular apoptosis, which led to an increase in scar formation. The observed scarring imitated human hypertrophic scars [185]. It was shown that the release of mechanical tension or a decrease in stiffness prevented the transition of the latent TGF-β1 form into the active one and caused a decrease in the α-SMA expression and contractility of mFBs [186].

The mechanical forces affecting the cells through mechanosensing cause changes in the cell phenotype and behavior (Scheme 1) [187,188]. The balance between extracellular forces acting on the cells (ECM or adjacent cells) and the response forces generated by the cells themselves is called tensional homeostasis [189]. This is necessary for proper cell function and efficient tissue remodeling. Fundamental and clinical studies of the cells and tissues mechanobiology emphasized the importance of mechanical forces in the process of skin development, regeneration, wound healing, and the pathogenesis of skin diseases [124,190,191,192]. The ability to regenerate lost tissue after injury is also determined by the local wound environment stiffness [191]. Tension plays an impotant role in wound healing and affects tissue repair [192].

## 9. Prevention of Contraction Facilitates Scarless Regeneration

Among mammals, a known example of scarless wound healing is skin regeneration in African Spiny mice, Acomys. The Acomys mice can regenerate up to 60% of the skin with glands and HFs. During the restoration of the spiny mice skin, a porous ECM rich in type III collagen is formed. In addition to the lack of induction of cytokines and chemokines, the ECM profile in Acomys mice skin wounds is closer to that of fetal wounds. Interestingly, after the restoration, the dorsal skin of African Spiny mice is weaker than that in normal mice [193,194], supposing that a softer surrounding matrix favors regeneration. Other examples of spontaneously healing skin wounds in different animal species with successful regeneration without a scar include skin wounds in the perforated rabbit ear [195,196,197,198], regeneration of the damaged oral mucosa in mice and pigs [199,200,201,202,203], and skin injuries in axolotl [204,205,206]. An analysis of studies where skin wounds in different animal species are restored spontaneously without scarring shows that the regeneration of these wounds coincides with the absence of their contraction [207]. Contraction is apparently an independent process that is not a component of regeneration. This is the primary reaction of the skin to a wound. It determines the healing process in the direction of scar formation, exerting a mechanical effect on the cells and the matrix of the wound bed synthesized by them. Contraction triggers the process of fibrosis and scarring, thus determining the properties of the regenerated tissue. Scar formation is critically dependent on wound contraction and, therefore, a healing process is secondary to the contraction. Regeneration in an adult mammal is suppressed due to the wound contraction, but not by the scar development. Recently, it has been demonstrated that regeneration of skin wounds and peripheral nerves in an adult mammal can be achieved simply through the appropriate control of wound reduction, rather than struggling against scar formation [207,208]. In these studies, a collagen scaffold (dermal regeneration template, DRT, modified Integra^®^) with certain characteristics (pore size, surface chemistry, and time of degradation) was used, which allowed mFBs to be removed from the pool of contractile cells by interacting with the scaffold. The pores were optimized to ensure the migration of the mFBs to the scaffold and provide a sufficient specific surface area for the interaction of cells with the scaffold; the α1β1 or α2β1 integrin ligands provide specific binding of mFBs on the surface of the scaffold. When the cell interacts with the scaffold, a dispersion of the stress fibers and randomization of the force vectors of the contractile cells occurs, which leads to a further decrease in macroscopic contractile force [207,208]. The half-life time is important for the scaffold’s regenerative ability. The premature onset of scaffold degradation abolishes its biological activity of binding contractile cells [209]. The long half-life time contributes to long-term inflammation and the presence of macrophages in the wound bed. When using DRT, the following phenotypic cell changes were observed: a significant decrease in the density of mFBs, dispersion of mFB assemblies, and loss of alignment of the axes of mFBs, which is associated with blocking the contraction and initiating the regeneration process. It is known that scar formation is also the result of collagen fiber alignment in the presence of the tensile stress field generated by a wound contraction process [210]. Yannas and colleagues presented evidence that an active scaffold with an optimal half-life time binding contractile cells eliminates a stretching field in a wound area and facilitates the formation of a physiologically new stroma instead of a scar, acting as a topographic framework for its synthesis [207].

In general, it is very important to take into account the characteristics of the scaffold to understand the biology of scar elimination and the development of scaffolds for SS that allow a high degree of wound regeneration. It turns out that monitoring and accounting for the area of wound reduction is not a valid criterion of successful wound healing, given that a high degree of contraction under implant application can have an impact on regenerative properties and contribute to scar formation. Therefore, it makes sense to consider the area of wound reduction by epithelialization.

Scar treatment with modulation of tissue tension (mechanomodulation therapy) is implemented in embrace^®^ Device, an FDA-approved device that minimizes tension on the skin and significantly improves aesthetic outcomes following surgical incisions and scar revision surgery [211,212]. It would be interesting to get long-term follow-up results from this product.

## 10. Skin Restoration Using Skin Substitutes

At the present time, a large number of SS with different characteristics have been developed [213]. In Table 2, we list the main criteria by which SS can be classified.

**Scheme 2:**
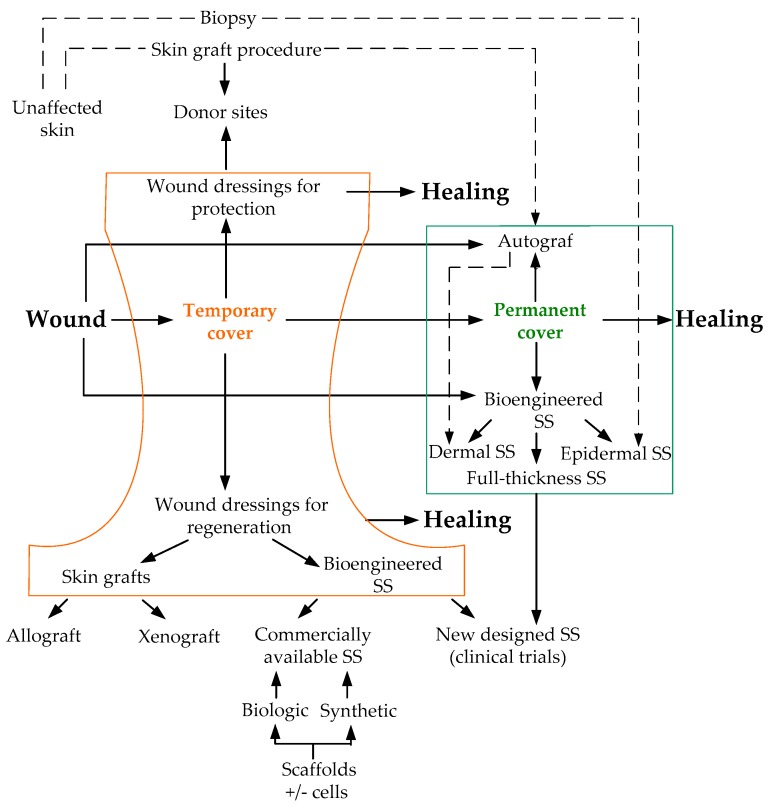
Wound dressings applicable for skin wounds. By permanent cover, we mean dressings which incorporate into patient’s regenerated skin. By temporary cover we mean dressings which provide protection and stimulation of healing. In case of extensive injuries, temporary covers are used to prepare the wound bed before autograft transplantation, or while a SS is prepared using autologous cells. We give one to two examples of each wound dressing/SS. Wound dressings for protection: amniotic membrane, films. SS as temporary cover: Biobrane, TransCyte. Allograft: cadaver skin graft. Xenograft: porcine skin graft. Full-thickness SS: Apligraf (in treating diabetic foot and venous ulcers). Dermal SS: Alloderm, Integra, then add ultrathin autologous skin graft. Epidermal SS: Epicell, Cell Spray. Biologic scaffold: HA, collagen. Synthetic scaffold: poly(lactic acid), poly(ethylene glycol). Hydrogels scaffolds are 3D hydrophilic polymeric networks. To cover donor cites, more expensive and complex bioengineered SS are used sometimes. SS—skin substitutes, HA—hyaluronic acid.

The classic full-thickness living SS is based on the co-cultivation of the dermal and epidermal cells in vitro. To seed keratinocytes on the dermis surface is not a difficult task, but the creation of the 3D dermis structure requires a special approach that includes the use of a scaffold. The scaffold plays the role of a supporting structure and ECM simultaneously; it is critical for the effective migration of cells from the dermis, the successful production of ECM, the further neovascularization, and ultimately, the timely degradation and proper formation of the newly formed tissue architecture. The scaffold must be biocompatible, biodegradable, non-toxic, non-inflammatory, and non-immunogenic. For mass scaffold use, the scaffold should be inexpensive and fast in production, easy to store, transport, and use. It is important that the use of a particular type of SS depends on the depth and area of damage as well as the wound pathogenesis and the characteristics of wound healing. In Scheme 2, we tried to represent the variants of wound dressings and the order of their application.

For example, before applying an autologous living SS as a permanent cover, an extensive wound needs to be closed with a temporary cover (Scheme 2), since the fabrication of autologous living SS takes about 14 to 28 days. The biodegradable temporary cover (if it is expected to persist in the wound bed for a long time) should integrate into the wound, promote FBs and other cell invasion, and new vessel formation from the wound bed. The created environment should contribute to the successful production of autologous collagen by the patient’s FBs. The temporary cover should prevent contraction, infection, be non-toxic, non-allergenic, and biotolerant, maintain a humid environment, and prepare the tissue until the permanent cover is transplanted. The neodermis formed within the SS framework must be flexible and slightly elastic, be able to withstand shear, and ensure wound stability for a long time [214]. The permanent cover must fit all of the criteria described above for the temporary cover as well as promote successful epithelialization to form high-quality regenerated tissue, maximally neutralize scar formation, and in theory, ensure the development of skin appendages.

According to current treatment standards, burn wounds are covered with autografts. The lack of a sufficient number of donor sites and the requirement for temporary coverage are problems that are currently being solved by trying to use the available SS. Any SS has its advantages and disadvantages [215]. Cadaveric allograft skin (cadaver skin) is still widely used in the treatment of burn wounds [216,217,218]. At the same time, researchers are trying to develop optimal SS that, in case of large damage areas, will replace the use of autografts and decrease the level of scar development.

For the treatment of wounds and scar incision, hydrogels have shown good results [219]. Recently, dextran-based hydrogel has been shown to contribute to complete skin regeneration with hair regrowth [220].

However, collagen remains the most widely used material as a scaffold. Collagen-based SS continues to be widely and actively studied, improved, and provides a satisfactory result in wound healing. Many are commercially available, and some are FDA approved [6,213,221,222]. Each of them, taking into account the advantages and disadvantages, seems to find its niche in wound healing. For example, collagen SS Integra^®^, (Integra Life Sciences), which has been used since the 1980s, has shown considerable versatility and has worked well in clinical settings [223]. However, the use of Integra^®^, like other SS, does not provide the regeneration of skin appendages, sebaceous, and sweat glands. This, in the case of damage to large areas of the body, causes great difficulties for patients.

In our study in mice, a rather rigid porous gelatin sponge did not favor wound healing and prevented the formation of granulation tissue, while a soft collagen gel populated with skin cells contributed to the reduction of the inflammatory process and effective wound healing: active granulation tissue vascularization, cell proliferation, remodeling, and rapid epithelialization [224]. Gelatin has previously been reported to be an inappropriate substrate for binding the contractile cells to the scaffold as unlike collagen, ligands on its surface bind poorly to collagen-binding integrins [207,225], although a hemostatic gelatin sponge is a suitable scaffold for osteogenic differentiation of preosteoblasts [226]. We observed that, at the onset of healing, keratinocytes migrated well into the porous structure of gelatin sponge, but the epithelial cells probably did not survive without the support of adequate granulation tissue. In addition, the porous structure of the gelatin sponge prevented successful long-term epithelialization, which does not allow cells to efficiently deposit basement membrane proteins [224].

The gelatinous sponge gave the worst wound healing result, most likely due to its unsuitable physical properties when compared to the collagen gel, which probably reduced tissue tension due to its structure and density. Our collagen SS enabled HF formation. At the same time, it should be considered that the model of a skin wound in rodents does not fully reproduce the process of human skin healing, which will be discussed below.

Currently, there have been no large-scale studies assessing the impact of SS on the subsequent quality of healing including scar formation and the full regeneration of the skin structure. There have been some studies on the long-term follow-up comparison of regenerated tissue after the application of different SS [227,228,229]. There are other studies dedicated to the investigation of the relevance of the dermal substitutes before the application of autologous split-thickness skin grafts (SSG). For example, on pigs, Philandrianos and colleagues came to the conclusion in a study of five acellular dermal SS that there were no long-term differences in scar quality between the different artificial dermal substitutes as well as with the control group only treated with autografts [230]. Using the Cutometer^®^, Nguyen and colleagues tested the hypothesis that after Integra^®^ application, scars were more pliable than patients’ SSGs by comparing the native patients’ skin with regenerated skin after the transplantation of Integra^®^ and skin grafts one year after treatment. The results showed that there were no differences between the Integra^®^, SSG sites, and the normal skin of the same patients [231].

When analyzing the results obtained by researchers in models of skin wound healing in animals, inevitably, the question arises of choosing a wound model in vivo and interpreting the short-term results of applying SS. As above-mentioned, it is known, for example, that a full-thickness splinted wound on a mouse back has a high mechanical tension, which increases the mFB activity, while, on the other hand, wounds on rabbit ears practically do not contract. At the same time, reliable models reflecting human hypertrophic scars have not been developed yet. There are already quite a lot of approaches and products that ensure the adequate closure of skin wounds. However, the effectiveness of these products is mostly not often evaluated in the long-term follow-up. Within this context, the criteria for such assessment are missing. In addition, given the accumulated knowledge in the field of wound healing and the diversity of SS types, it is apparently time to introduce such criteria. There is a need to analyze data of extensive and successful experiences of using various SS/scaffolds for skin wound healing and their comparative analysis in terms of clear characteristics. Both the immediate and long-term results of treatment should be evaluated, including “regenerative” qualities such as structure, barrier properties, and vascularization as well as the risks of infection, rejection, relapse, scar formation, restoration of skin appendages, etc. Such analysis will provide a clear understanding of which tasks a particular product is best suited for. Newly developed products could be evaluated according to this scheme. A similar approach will help to determine “the golden standard” in choosing the most effective SS for regeneration. For proper and complete skin regeneration, we need to move toward the new generation of SS. It will be extremely useful to develop SS that takes into account the dermal FB heterogeneity and impact of other cell types including endothelial cells, HF cells, preadipocytes, neuronal cells, melanocytes, etc. Cells within SS possess their own biological activity including ECM modification. As discussed above, the use of a certain subpopulation of FBs, for example, may significantly alter the influence of SS on the wound healing process. Advances in HF reconstruction [37,45,46,48,232,233] and skin-on-chip solutions [234,235,236] can provide the key to the problem of the high-quality regeneration of skin structure including appendages.

The development of these approaches, taking into account the factors contributing to fibrosis, is likely to achieve minimal scarring and good cosmetic results.

In the field of studies to prevent or reduce skin scarring and achieve a maximally natural structure of the regenerated skin, the following directions can be pointed out: (1) the transfer of the dermis to the “embryonic-like” status; (2) papillary dermis activation; (3) neogenesis of skin appendages in the area of damage; (4) WNT signaling pathway regulation; (5) regulation of the ECM protein synthesis, metalloproteinase activity, and signaling molecule release; (6) mFBs inactivation; (7) release of mechanical tension and tissue contraction leveling; and (8) application of SS scaffolds which contribute to wound relaxation, provide proper cell distribution, reduce contraction and scar formation.

## 11. Conclusions

In this review, we discussed the heterogeneity of the dermis and FBs that inhabit it, with an emphasis on their contribution to wound healing and scar formation. Apparently, wound repair in the modern world involves not just its closure in the form of a patch, but the reconstruction of a mostly identical structure, restoration of function, and aesthetics. Previously, the scar was mainly considered as the deposition of pathologic ECM and an inevitable outcome of wound healing in vertebrates including humans. At present, vast knowledge has been accumulated concerning diversity of dermal cells and pathways that regulate scar formation, enabling one to think about its significant reduction, or even elimination, using up to date SS comprising proper scaffolds and cell types. The relaxation of tissue tension is thought to be a significant, if not the key aspect, of eliminating scar development. Novel SS considering these points can provide a high quality of regenerated tissue.

## Figures and Tables

**Table 1:** Characteristics of papillary versus reticular FBs.

Characteristics	Papillary FBs	Reticular FBs
Morphology	Spindle-shaped [11]	Strongly spread out over the substrate and have a stellate shape [22]
Extracellular matrix	Weaker alignment of collagen fibers [5], a higher content of proteoglycans [4]	Ordered network of collagen fibers and elastin strands [7]
Positivity for α-SMA	Small part of FBs is α-SMA positive [13]	Most part of the FBs is α-SMA positive [13,19].
Functional activities in culture	High proliferative and synthetic activity [12,13,17]	Low proliferative and synthetic activity [12,13,17]
Expression of cell markers in mice dermis	Express genes of the WNT pathway proteins and CD39 [11]; CD26+Sca1–FBs express COL6A5, COL23A1, APCDD1, HSPB3, CD3g, CD3d, CD3ε [11]	Include Dlk1+Sca1+ and Dlk1–Sca1+ preadipocytes [11]; Dlk1+Sca1−FBs express genes of the secretoglobin superfamily [11], CD36 is upregulated in the lower reticular FBs [11]
Expression of cell markers in human dermis	Express genes of the WNT pathway proteins and CD39 [11]; FAP+CD90–FBs express PDPN and NTN1 [13,16]	FAP−CD90+ FBs express high levels of ACTA2, MGP, PPARγ, CD36 [16]; FBs express CD26, MFAP5, PRG4 [11], MGP and TGM2 [13]
Contribution to skin equivalents	Support the formation of a multilayered, more stratified and differentiated epidermis with epidermal ridges [3,11]. ECM components promote the predominant growth of keratinocytes [21]	Do not support the formation of fully-stratified epidermis [11]. The composition of the basement membrane is altered [23]
Scarring	May improve scar condition [30]	Generate the matrix typical for fibrosis [14]

**Table 2:** Criteria for skin substitutes classification.

**Type of scaffold**	Biologic Biosynthetic Synthetic
**Substitute thickness**	Split-thickness Full-thickness
**Number of layers**	Single-layered Bi-layered Multi-layered
**Cell origin**	Autologous Allogenic Xenogenic Acellular
**Cell type included**	Keratinocytes Fibroblasts Other cell types
**The skin layer to be replaced**	Epidermis Dermis Compound
**Period of application**	Temporary Permanent
**Type of wound to be treated**	Superficial Deep
**Prospective “regenerative” criteria**	Effect on contraction Vascularization Epithelialization rate Scar parameters The ability to stimulate skin appendages regeneration Long-term follow-up
